# Control strategies used in lower limb exoskeletons for gait rehabilitation after brain injury: a systematic review and analysis of clinical effectiveness

**DOI:** 10.1186/s12984-023-01144-5

**Published:** 2023-02-19

**Authors:** Jesús de Miguel-Fernández, Joan Lobo-Prat, Erik Prinsen, Josep M. Font-Llagunes, Laura Marchal-Crespo

**Affiliations:** 1 Biomechanical Engineering Lab, Department of Mechanical Engineering and Research Centre for Biomedical Engineering, Universitat Politècnica de Catalunya, Diagonal 647, 08028 Barcelona, Spain; 2 Institut de Recerca Sant Joan de Déu, Santa Rosa 39-57, 08950 Esplugues de Llobregat, Spain; 3 ABLE Human Motion, Diagonal 647, 08028 Barcelona, Spain; 4 Roessingh Research and Development, Roessinghsbleekweg 33b, 7522AH Enschede, Netherlands; 5 Cognitive Robotics Department, Delft University of Technology, Mekelweg 2, 2628 Delft, Netherlands; 6 Motor Learning and Neurorehabilitation Lab, ARTORG Center for Biomedical Engineering Research, University of Bern, Freiburgstrasse 3, 3010 Bern, Switzerland; 7 Department of Rehabilitation Medicine, Erasmus MC University Medical Center, Doctor Molewaterplein 40, 3015 GD Rotterdam, The Netherlands

**Keywords:** Powered exoskeleton, Gait rehabilitation, Lower limb, Brain injury, Stroke, Cerebral palsy, Literature synthesis

## Abstract

**Background:**

In the past decade, there has been substantial progress in the development of robotic controllers that specify how lower-limb exoskeletons should interact with brain-injured patients. However, it is still an open question which exoskeleton control strategies can more effectively stimulate motor function recovery. In this review, we aim to complement previous literature surveys on the topic of exoskeleton control for gait rehabilitation by: (1) providing an updated structured framework of current control strategies, (2) analyzing the methodology of clinical validations used in the robotic interventions, and (3) reporting the potential relation between control strategies and clinical outcomes.

**Methods:**

Four databases were searched using database-specific search terms from January 2000 to September 2020. We identified 1648 articles, of which 159 were included and evaluated in full-text. We included studies that clinically evaluated the effectiveness of the exoskeleton on impaired participants, and which clearly explained or referenced the implemented control strategy.

**Results:**

(1) We found that assistive control (100% of exoskeletons) that followed rule-based algorithms (72%) based on ground reaction force thresholds (63%) in conjunction with trajectory-tracking control (97%) were the most implemented control strategies. Only 14% of the exoskeletons implemented adaptive control strategies. (2) Regarding the clinical validations used in the robotic interventions, we found high variability on the experimental protocols and outcome metrics selected. (3) With high grade of evidence and a moderate number of participants (N = 19), assistive control strategies that implemented a combination of trajectory-tracking and compliant control showed the highest clinical effectiveness for acute stroke. However, they also required the longest training time. With high grade of evidence and low number of participants (N = 8), assistive control strategies that followed a threshold-based algorithm with EMG as gait detection metric and control signal provided the highest improvements with the lowest training intensities for subacute stroke. Finally, with high grade of evidence and a moderate number of participants (N = 19), assistive control strategies that implemented adaptive oscillator algorithms together with trajectory-tracking control resulted in the highest improvements with reduced training intensities for individuals with chronic stroke.

**Conclusions:**

Despite the efforts to develop novel and more effective controllers for exoskeleton-based gait neurorehabilitation, the current level of evidence on the effectiveness of the different control strategies on clinical outcomes is still low. There is a clear lack of standardization in the experimental protocols leading to high levels of heterogeneity. Standardized comparisons among control strategies analyzing the relation between control parameters and biomechanical metrics will fill this gap to better guide future technical developments. It is still an open question whether controllers that provide an on-line adaptation of the control parameters based on key biomechanical descriptors associated to the patients’ specific pathology outperform current control strategies.

**Supplementary Information:**

The online version contains supplementary material available at 10.1186/s12984-023-01144-5.

## Background

Brain injury is a wide open concept associated with damage to the brain due to events inside of the body, i.e., non-traumatic brain injuries, or external forces, i.e., traumatic brain injuries (TBIs). Non-traumatic brain injuries include stroke or cerebral palsy. Brain injuries are one of the major causes of death and disability worldwide [1]. The global incidence of stroke increases by more than 13.7 million new cases each year [2], and is the third leading cause of disability worldwide [3]. The prevalence of cerebral palsy is estimated to be from nearly 2 to nearly 3 per 1000 newborns worldwide [4, 5]. Traumatic brain injury is another leading cause of disability around the globe, with 69 million survivors every year [6].

Difficulty in standing and walking is one of the major consequences of brain injuries. For instance, over 63% of stroke survivors suffer from half-mild to severe motor and cognitive disabilities [7], and 30–36% are unable to walk without assistive aids [8, 9]. This results in loss of independent mobility and limits community participation and social integration, which causes secondary health conditions [10]. Individuals with brain injuries can exhibit common motor impairments, like paralysis, spasticity, or abnormal muscle synergies, leading to compensatory movements and gait asymmetries [11–15]. This pathological gait hinders a skilful, comfortable, safe, and metabolically efficient ambulation [16].

The recovery process after a brain injury takes months to years and neurological impairments can be permanent [17]. There is strong evidence that early, intensive, and repetitive task- and goal-oriented training, which is progressively adapted to the patients level of impairment and rehabilitation stage, can improve functional ambulatory outcomes [11, 18–23]. However, due to limited resources and the heterogeneity of impairment, it is challenging for physiotherapists to provide the required intensity and dose of training, while extracting quantitative information to maximize functional walking ability for a specific patient.

Robotics can play a promising role in gait rehabilitation for individuals with brain injuries. Robots allow performance of wide range of tasks—e.g., walking, sitting up/down, or walking on a slope—with high intensity. Some robotic controllers might also promote patients’ active participation and engagement during the training process, e.g., by varying the level of the assistive force [24, 25]. High repeatability and intensity of training, together with patients’ engagement, have been listed as crucial factors to induce neural plasticity and motor learning [26–28]. Importantly, clinical evidence suggests that combining robotic and conventional rehabilitation training positively impacts the ability to walk independently, walking speed, and walking capacity, although there is still no solid evidence about the superiority of robotic rehabilitation over conventional therapy [29–33].

Lower-limb exoskeletons promote task-oriented repetitive movements, muscle strengthening, and movement coordination, which have been shown to positively impact energy efficiency, gait speed, and balance control [34, 35]. Exoskeletons, compared to other robotic solutions, e.g., patient-guided suspension systems and end-effector devices, allow for full control of the leg joint angles and torques, and are the preferred robotic solutions for training brain-injured patients who suffer from severe motor disabilities [36]. Thereby, we consider that focusing on exoskeleton technology is a wide and rich enough topic to extract conclusions on the clinical effectiveness of the control strategies in the broad group of brain-injured patients [37–39].

The interest on lower-limb exoskeletons for gait rehabilitation has increased exponentially in the last years, which is reflected in the considerable number of reviews published within the last decade [38, 40–60]. However, the majority of these reviews focus on hardware, while only a few of them analyzed the control strategies implemented on lower limb exoskeletons and their effects on walking function in individuals with brain injuries [38, 41, 42, 54–60]. Yet, the control strategy—as ergonomics and robot actuation—might play a key role on the effectiveness of the robotic treatment [61]. As in every biological system, control rules are essential to modulate every action attending to internal and external factors [62].

We found a few literature surveys that focused on control strategies for lower-extremity exoskeletons: Baud et al. and Li et al. categorised the control strategies and actuation systems implemented on lower-limb exoskeletons [41, 42]; Chen et al. presented a review on wearable hip exoskeletons for gait rehabilitation and human performance augmentation that addressed actuation system technologies and control strategies [57]; Zhang et al. presented a review on lower-limb exoskeletons offering details about actuation systems, high-level control, and human–robot synchronization tools [38]; Tucker et al. [55] reviewed several control strategies, gait pattern recognition, and biofeedback approaches for lower extremity robotic prosthetics and orthotics. Finally, a recent systematic review on wearable ankle rehabilitation robots for post-stroke rehabilitation focused on actuation technologies, gait event detection, control strategies, and the clinical effects of the robotic intervention [59].

In this systematic review, we aim at complementing previous literature surveys by providing an updated structured framework of current control strategies, analyzing the methodology of clinical validations used in the robotic interventions, and reporting the potential relation between the employed control strategies and clinical outcomes. In this literature survey we seek to answer the following three research questions: (1) Which control strategies have been used on powered lower limb exoskeletons for individuals with brain injuries?, (2) What are the experimental protocols and outcome metrics used in the clinical validation of robotic interventions?, and (3) What is the current clinical evidence on the effectiveness of the different control strategies?

## Methods

### Search strategy

To answer the first research question—i.e., which control strategies have been used on powered lower limb exoskeletons for individuals with brain injuries?—we conducted a literature search on the 17th of September 2020, including English-language studies published from January 2000 to September 2020 in four databases: Web of Science, Scopus, PubMed, and IEEE Xplore. The search included the following keywords: (“brain injury” OR “cerebral” OR “palsy” OR “stroke” OR “hemipare*” OR “hemiplegi*” OR “CVA” OR “cerebrovascular accident” OR “cerebral infarct” OR “cerebral hemorrhage” OR “ABI” OR “acquired brain injury” OR “motor learning” OR “neuroplasticity” OR “neural plasticity” OR “neuroplastic”) AND ((“lower” AND (“limb*” OR “extremit*”)) OR “walk*” OR “ambulat*” OR “gait”) AND (“power*” OR “active” OR “robot*” OR “wearable”) AND ( “assistive” OR “exo*” OR “exosuit” OR “exo-suit” OR “brace*” OR “ortho*”) AND “control*”.

**Fig. 1:**
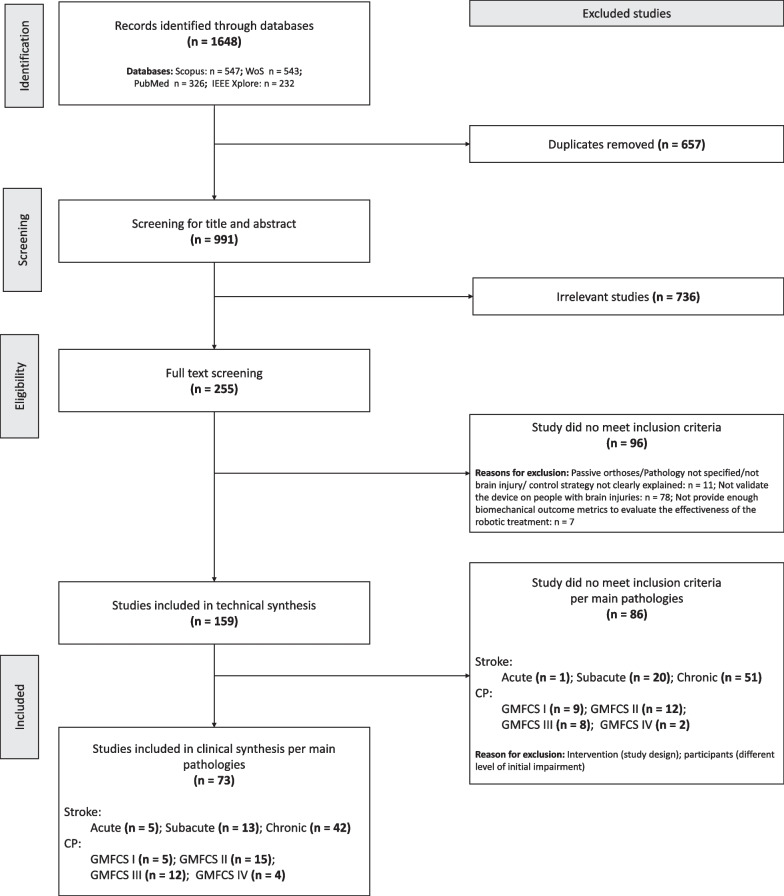
PRISMA flowchart for identification and screening of eligible studies for the current review. The total number of studies is not equal to the sum of the studies divided per level of impairment and/or acuity as, in some studies, participants were pooled together independently of their acuity or GMFCS levels

The search query led to 1648 studies (991 after removing duplicates). After a title and abstract screening, the number of studies was reduced to 255. Then, a full-text screening process was carried out with the following criteria: studies should (1) involve active orthoses/exoskeletons for lower-limb training, (2) provide technical details about the control strategy used, (3) validate the device on individuals with a brain injury, and (4) report biomechanical or clinical outcome metrics that allow for a comparison among different control strategies. The last condition was associated with the analysis of the clinical methodology followed in robotic interventions. After the full-text screening, a total of 159 publications were included in this review (see Fig. 1), with a total of 43 different lower limb exoskeletons. The resulting studies will be used to answer the first two research questions outlined in this review. See Additional file 1 for a detailed list of the studies included.

### Clinical comparison

To answer the third research question—i.e., what is the current clinical evidence on the effectiveness of the different control strategies?—we conducted a stricter screening of the 159 publications focusing on the studies that performed an assessment before and after the robotic intervention; the studies that focused only on assessments *during* the robotic intervention while wearing the robotic device or only immediately after a single training session were not included (see Fig. 1).

To perform an *unbiased* clinical comparison between different exoskeleton controllers, we subdivided the individuals with stroke and CP into different subgroups, based on their impairment level and/or acuity before the robotic intervention. For the stroke group, we used three levels of acuity: acute (≤ 2 weeks from stroke onset), subacute (≤ 6 months from stroke onset), and chronic (> 6 months from stroke onset). In the case of CP, we followed the four levels of the Gross Motor Function Classification System (GMFCS) [63].

Applying a final screening process, we only compared controllers tested with participants who shared similar levels of impairment before the robotic treatment, i.e., similar scores in Functional Ambulation Category (FAC) and in the metrics mentioned in “Outcomes of interest for the clinical comparison” section. This resulted in the exclusion of six studies on individuals with acute [64], subacute [64–69], and chronic [69] stroke.

This final screening process led to 73 studies of which 57 studies included stroke survivors (78.08% of the studies) and 16 children/adults with CP (21.91% of the studies). From the 57 studies that analyzed the benefits of robotic exoskeleton lower-limb training on stroke survivors, five studies included participants with acute stroke, 13 studies with subacute stroke, and 42 studies with chronic stroke. From the 16 studies with children/adults with CP, five studies included participants with GMFCS I, 15 studies with GMFCS II, 12 studies with GMFCS III, and four studies with GMFCS IV. Note that the total number of studies is not equal to the sum of the studies divided per level of impairment and/or acuity as, in some studies, participants were pooled together independently of their acuity and GMFCS levels. See Additional file 2 for a detailed list of the studies included in the clinical analysis.

To further analyze, compare, and discuss the effectiveness of different control strategies, we also took into consideration: (1) the grade of evidence based on the type of intervention—e.g., (randomized) clinical trials or observational studies, (2) the training intensity of the robotic treatment—i.e., the product of the session duration, number of sessions, and frequency of the training, and (3) the number of participants who trained with each type of control.

Following the guidelines presented in [70, 71], we considered that a study had a high *level of evidence* (level I study) when it was a Randomized Clinical Trial (RCT). When the study was a Clinical Trial (CT), we considered that its level of evidence was moderate (level II study). Finally, the level of evidence of observational studies was considered low (level III study). The *grade of evidence* of the clinical effects of the robotic treatment was considered strong when there was a preponderance of level I and/or level II studies that supported the result—this must include at least one level I study. The grade of evidence was considered moderate when there was a preponderance of level II and/or level III studies that supported the result—this must include at least one level II study. Finally, the evidence was classified as weak when only level III studies supported the result.

#### Outcomes of interest for the clinical comparison

The selection of the outcome measures of interest was based on those recommended by surveys and studies that evaluated stroke and CP rehabilitation [72–78]. To evaluate the effectiveness of the control methods on stroke survivors, we selected the following scales: Berg Balance Scale (BBS), 10 m Walk Test (10MWT), 6 min Walk Test (6MWT), Timed-Up and Go (TUG), Fugl–Meyer Assessment of Lower Extremity (FMA-LE), and Functional Independence Measure (FIM)—this last one only for acute stroke. To evaluate the effectiveness of the control strategies on individuals with CP, we selected the following scales: Gross Motor Function Measure (GMFM)-66/88 dimensions D and E, 10MWT, and 6MWT.

### Control strategies taxonomy

**Fig. 2:**
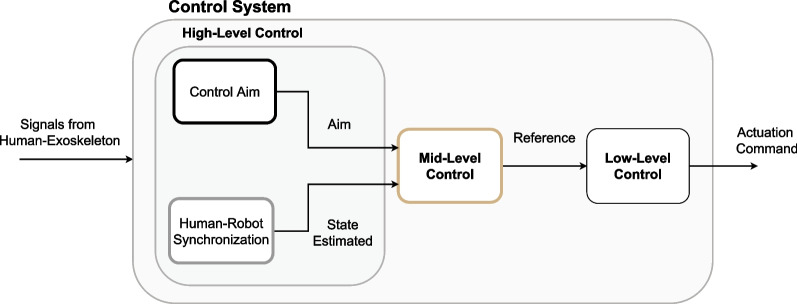
General control system diagram. The *signals from the Human–Exoskeleton*—e.g., human–robot interaction forces, limbs’ kinematics, and/or recorded human muscle or brain activity—are processed sequentially by three different blocks—each corresponding to High-, Mid- and Low-level control, to generate the *actuation command*. High-Level Control: the *Control Aim* defines the role of the exoskeleton in the overall performance of the human-exoskeleton system, i.e., enhance or hinder task completion. The *Human–Robot Synchronization* block generates an estimation of the actual state and is used by the Mid-level control, together with the control aim, to provide reference values—e.g., desired position or force—to the Low-level control. The Low-level Control then transforms that reference into actual assistive/resistive force/motion and sends the actuation command to the exoskeleton hardware

To analyze the state of the art of control strategies for lower limb exoskeletons in rehabilitation, we propose a hierarchical classification of control methods based on an adapted version of the categorization presented in [55]. The hierarchy establishes three different levels: High-level control, Mid-level control, and Low-level control (see Fig. 2).

High-level controllers are defined as control strategies that identify the human’s volitional intent and select the appropriate exoskeleton response behaviour. The exoskeleton Mid-level control reacts to the current state of the user and defines the reference position or force that the robot should follow based on the control aim and the state estimated by the human–robot synchronization algorithm (both embedded in the High-level control) and the sensors measurements. Finally, the Low-level control tries to achieve the desired state determined by the Mid-level controller by applying feedforward or feedback control. In this systematic review, we have focused on High- and Mid-level controllers since they are highly related to exoskeleton use, while Low-level controllers are directly linked to the hardware and can be applied in other types of robots [41].

#### High-level control

**Fig. 3:**
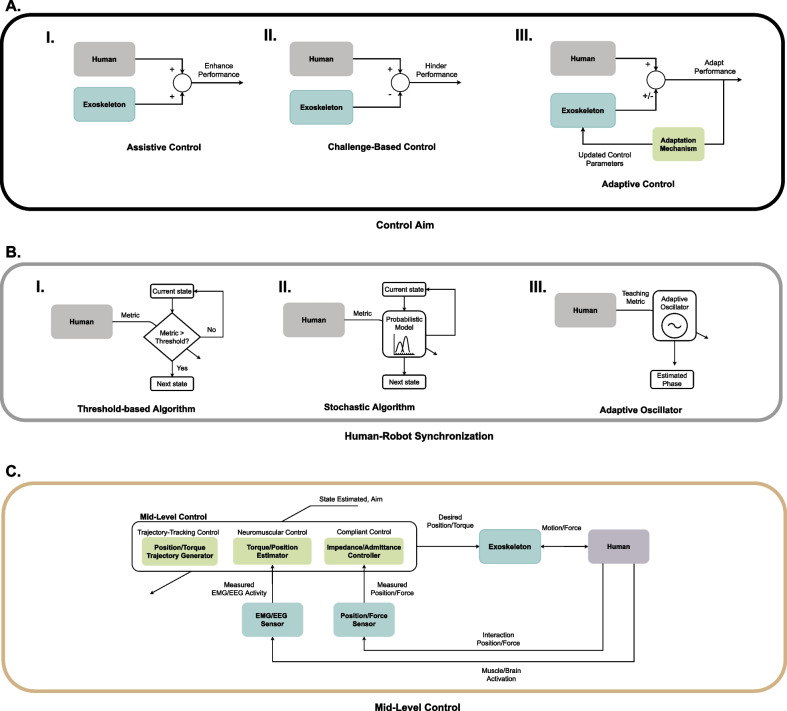
Taxonomy of High- and Mid-level controllers. **A** Control Aim: **AI** In *Assistive Control*, the exoskeleton provides support to enhance the movement performance during training. **AII** Conversely, in *Challenge-Based Control* mode, the exoskeleton provides actions that hinder the human performance. **AIII** *Adaptive Control* adjusts the system parameters based on the human–robot performance to provide adjusted assistance or resistance. **B** Human–Robot Synchronization: **BI** *Threshold-Based Algorithms* ensure the transition between states whether the detection metric fulfils a pre-defined threshold. **BII** In *Stochastic Algorithms*, the transition between states for the same set of initial conditions and algorithm parameters might be different due to the inherent randomness of the models used. **BIII** *Adaptive Oscillators* use the periodic motion of the patient to extract its phase either to generate a control signal or to determine the actual state of the patient, e.g., the phase of the gait. **C** We categorize the Mid-level control strategies used in lower-limb exoskeletons for gait rehabilitation into three families: *Trajectory-Tracking Control* generates reference assistive or resistive torque/position profiles based on parameterized or pre-recorded position/torque trajectories; *Neuromuscular Control* uses recorded biosignals (e.g., brain/muscle signals) to generate the control signal for the Low-level control; and *Compliant Controllers* regulate the impedance or admittance of the exoskeleton by modifying the dynamic relation between movement and force or force and velocity, respectively

A High-level control system provides a command that modifies the state of the actuation system according to the control aim [79–81] (see Fig. 3A). The **Control Aim** varies the purpose of the exoskeleton based on the desired treatment approach, e.g., assists or challenge the patients.

*Assistive* High-level controllers facilitate functional training by supporting the patients' movements to complete the task—e.g., sit-to-stand [82], achieve stability during the loading response of the gait [83], or plantarflexion assistance in late stance [84]. Assistance can be provided while patients are fully guided by the exoskeleton and remain passive during the training—i.e., haptic demonstration [81], or while patients actively execute the task while they are guided/corrected by the robot—i.e., haptic assistance [81]. It is thought that guiding movements while patients remain passive may improve gait performance [85–87], especially in those suffering from severe impairment [55, 88]. Additionally, mobilizing the affected limbs while patients remain passive allows for stretching the muscles and might reduce spasticity [89], provides somatosensory stimulation that facilitates restoring normative patterns of motor output [87], and importantly, provides an environment for safe, high intensity, and motivating locomotion training.

On the contrary, *Challenge-based* High-level controllers aim at, e.g., strengthening the muscles by opposing to task completion—e.g., resistive methods [90], enhancing error detection—e.g., error augmentation methods [91], and increasing movement variability—e.g., perturbation methods [92]. These challenge-based control strategies might lead to improvements in physical performance, movement control, walking speed, and functional independence, especially in people in the late stages of the rehabilitation or with mild impairment [93–96].

*Adaptive control* strategies aim to modify the control parameters based on the patient's specific needs [97]. In general, the control parameters of the exoskeletons have to be tuned to properly adapt to each specific patient's walking capabilities, as they are not generalized enough to capture the heterogeneity of gait disorders [98, 99]. It has been found that when setting up the exoskeleton, tuning the control parameters, together with donning, requires the highest amount of time [98, 100, 101]. Tuning is a laborious process, as therapists must manually modify the parameters relying only on subjective feedback from the patients and visual assessments of the gait pattern [99, 102]. A potential solution to guide the physiotherapists through the tuning process might be to provide an initial set of parameters that has been automatically tuned off-line based on the users’ baseline performance [99, 103]. However, automatic off-line or manual tuning might lead to a suboptimal set of parameters, which does not take advantage of the full potential of the exoskeleton to improve the rehabilitation effect [98]. Therefore, strategies that automatically adapt the control parameters of the exoskeleton in real-time, e.g., based on the patient's performance, could increase the positive effect of the exoskeleton while enhancing its usability by reducing the time needed to tune the control parameters.

Synchronization to the user’s motion is a key factor to effectively benefit from the exoskeleton therapy, e.g., reducing adaptation time and metabolic rate [104]. Most of the Mid-level control strategies need an estimation of the current action performed by the user to properly assist or resist her/his motion, i.e., to synchronize the human and the robot. The **Human–Robot Synchronization** sub-level within the High-level control estimates the state of the patient by using deterministic or stochastic methods based on recorded kinematic, kinetic, and/or bioelectric data—e.g., joint kinematics [105], ground reaction forces [106], human–robot interaction forces [107], muscular activity [108], and brain activity [109] (see Fig. 3B).

*Threshold-based algorithms* differentiate between states—e.g., gait phases [110], falling [111], and start-stop walking [112]—following a state-machine structure that allows the transition between states depending on logical rules.

*Stochastic algorithms*, on the other hand, infer the state throughout statistical models, e.g., using Linear Discriminant Analysis (LDA) [109], Hidden Markov Models [113], Principal Component Analysis [114], K-Nearest Neighbours [115], or Neural Networks [116]. This family of human–robot synchronization methods is particularly useful for planning the gait pattern of the exoskeleton based on vision-based environment classification due to the high performance of stochastic algorithms to classify environments using images [117].

Bio-inspired models are emerging as an alternative to threshold-based and stochastic algorithms. For example, *adaptive oscillators* are non-linear models that synchronize with a teaching signal—e.g., the thigh angle in the sagittal plane [118]—in phase, frequency and amplitude, mimicking bio-inspired behaviours [119]. The estimated output from the adaptive oscillator—e.g., phase of the input signal—is used to estimate the phase of the gait or to generate reference joint trajectories to assist or resist the human motion [118, 120, 121]. The main disadvantage of adaptive oscillators, however, is that they require precise parameter tuning to quickly synchronize with the human periodic motion [122].

Nevertheless, all human–robot synchronization methods require a parameter tuning to properly adapt to each specific patient's gait as they are not generalizable enough to avoid patient-to-patient variability [98]. This process is laborious, as therapists must manually tune the parameters off-line relying only on feedback from the patients and subjective visual assessments [99, 102]. Automatic adaptation [123] based on the patient's intention and/or gait parameters, such as gait speed [124–126], might facilitate the usability of these methods.

#### Mid-level control

Mid-level control employs sensor measurements, the control aim, and the state inferred by the human–robot synchronization to generate reference control commands used by the Low-level control to apply the actuation command (see Fig. 2). Three different families of Mid-level control strategies can be distinguished depending on the control inputs/outputs and controllers employed (see Fig. 3C).

*Trajectory-tracking control* generates predefined *position* or *force* trajectories as reference commands to provide assistance/resistance. These trajectories are usually determined based on pre-recordings of unimpaired individuals (e.g., hip and knee flexion-extension, and ankle plantarflexion-dorsiflexion torques [127]), information from the non-paretic limb (e.g., hip and knee flexion-extension angles [105, 128]), or pre-recorded trajectories during therapist-guided assistance (e.g., foot trajectory [129] or knee flexion-extension [130]).

*Neuromuscular control* strategies use biosignal recordings as control signals to decode the actions of the patient and send reference values to the Low-level control [131]. Common approaches, like myoelectric [132–134] and Brain-Computer Interface (BCI) [135, 136] control, use muscular—electromyography (EMG)—and brain—electroencephalography (EEG)—signals, respectively, to handle the control objective.

Lastly, *compliant controllers* [137, 138] regulate the impedance [113, 139] or admittance [140, 141] levels of the exoskeleton by modifying the dynamic relation between movement and force or force and velocity, respectively, using virtual dynamics of springs, dampers, or masses. The combination of trajectory-tracking control [142] or neuromuscular control [143] with compliant control usually provides a more flexible behavior to the exoskeleton during rehabilitation—e.g., by allowing more movement variability around the desired trajectory, compared to conventional rigid Low-level controllers such as proportional-derivative (PD) controllers [144, 145].

#### Neuroscience evidence behind current control developments

Neuroscience evidence seems to indicate that the aim of the control strategy of an exoskeleton for individuals with brain injuries should be to stimulate physical/cognitive engagement and motor learning rather than enforce repetitive movements with low variability [21, 146–148]. For this reason, control strategies for individuals with brain injuries should guarantee the patient’s active physical and cognitive engagement by providing tailored and compliant assistance or resistance. In particular, in individuals with moderate/mild brain injuries, excessive assistance may have a negative influence on motor learning, as the dynamics of the task to be learned is different from the trained task [149]. To promote patient's active participation, the device should engage the users wearing the exoskeleton to, e.g., actively initiate each step, inter-joint coordination or control their balance. This can be achieved by, e.g., adjusting the level of assistance or resistance based on real-time biomechanical measurements during locomotion. Thus, non-compliant generic controllers [104] that do not adapt their assistance/resistance might not be the most effective ones for gait rehabilitation of individuals with brain injuries who preserve partial or full volitional control [14, 147, 150]. Robotic training using controllers that modulate the assistance based on patient's performance or that allow for more compliant human–robot interaction might be more effective to stimulate motor learning than those that enforce generic “normative” movements independently of the patients' capabilities [151].

## Review

### Implementation of control strategies

**Fig. 4:**
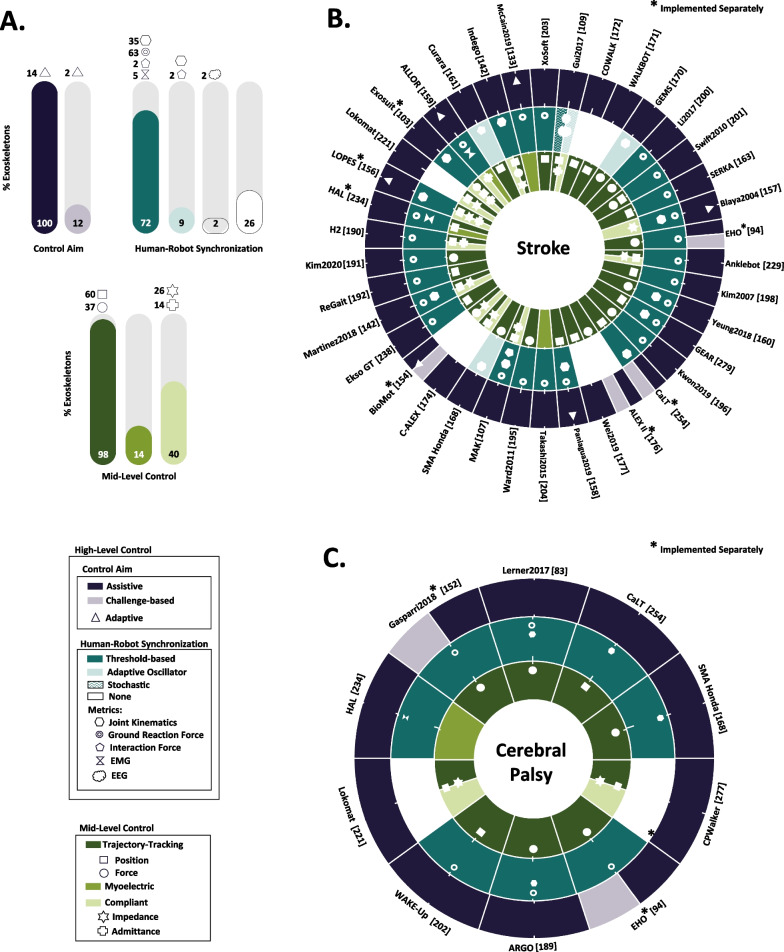
Overview of exoskeletons based on their High- and Mid-level control strategies. Each color represents the different families inside the High- and Mid-level controllers and the symbols point out the categories inside these families. **A** Percentage of exoskeletons that implemented the different families and categories of High- and Mid-level controllers for all the pathologies included in this review. Note that the same exoskeleton could incorporate different controllers, and therefore, the summation of percentages can be higher than 100%. **B**, **C** Circular plots illustrate the High- and Mid-level control strategies of exoskeletons tested on individuals with stroke (**B**) and cerebral palsy (**C**). Each circular sector represents a different exoskeleton and every ring represents different levels of the control hierarchy. The outer ring is the control aim, the middle ring is the human–robot synchronization, and the inner ring is the Mid-level control. If a symbol lies in the middle of a subdivision within a sector, it implies that the characteristic related to that symbol applies to both subdivisions

In this section we provide an overview of the High and Mid-level control strategies implemented in the studies included in this review from a technological point of view, without focusing on clinical aspects (see Fig. 4A). Exoskeletons used with individuals with stroke and cerebral palsy are highlighted as these two were the most predominant pathologies in the reviewed studies (see Fig. 4B, C).

#### High-level control: control aim

All of the exoskeletons validated on stroke survivors and children/adults with cerebral palsy implemented assistive strategies. On the other hand, only 10.5% of the exoskeletons for stroke rehabilitation and 20.0% for cerebral palsy validated challenged-based control strategies, e.g., using resistive forces [152–154], perturbing forces [92], or haptic error augmentation [155]. Note that the same exoskeleton could incorporate different controllers, and therefore, the summation of percentages can be higher than 100%.

Notably, only 14% of the exoskeletons used adaptive assistive control strategies and 2% used adaptive resistive control strategies. The parameters of the exoskeleton were automatically adapted in real-time based on real-time measurements of the patient’s biomechanics, e.g., the ankle angle tracking error [154, 156], hip and knee kinematics [98], gait speed [157, 158] or vertical ground reaction force [159]. The rest of the devices automatically or manually tuned the magnitude of the assistance off-line based on the patient’s motor function, previously assessed by therapists [98, 99, 160].

The lack of studies that adapted the assistance or resistance based on direct gait biomechanical descriptors of the brain-injured population might be due to the small number of reviewed studies that analyzed the effect of the control parameters on the patients' gait kinematics and kinetics [103, 156, 161–164]. Besides, the majority of these few studies only focused on analyzing the effect of the timing and magnitude of the assistive torque or position trajectories on ankle power [103], walking speed, step length, joint kinematics [161, 163, 164], metabolic cost, or muscular activity [162]. Only one study explored the effect of varying the parameters of an impedance model on the ankle position on the sagittal plane [156]. Yet, biomechanical metrics—e.g., step length [165], hip hiking [166], and trailing-limb angle during the stance phase [167]—might more directly reflect the patients' rehabilitation progress. Thus, control strategies based on these descriptors might increase the rehabilitation effect of the exoskeleton in comparison to non-adaptive strategies.

#### High-level control: human–robot synchronization

Threshold-based approaches were the most implemented human–robot synchronization algorithms on lower-limb exoskeletons for individuals with brain injuries in general (72.1% of the exoskeletons), and stroke survivors (73.6% of the exoskeletons) and cerebral palsy participants (80.0% of the exoskeletons) in particular.

Adaptive oscillators were tested with individuals with stroke in four different exoskeletons (10.5% of the exoskeletons) using sagittal lower-limb segment angles, joint angles, or robot–human interaction forces as synchronization signals [161, 168–170].

A few number of devices (25.6%) did not implement any type of event detection algorithm for human–robot synchronization, probably because they did not strictly need it [171–177]. Most of them were grounded exoskeletons that either enforced joint angle reference trajectories during gait—based on the unimpaired joint movement—using assistive control strategies [171, 172], or employed an assistive controller around the desired trajectory [173–177].

Only one exoskeleton in this review implemented stochastic methods to distinguish between different locomotion modes, i.e., stop, normal walk, acceleration, and deceleration [109]. They used linear discriminant analysis (LDA) with EEG signals to differentiate between the frequencies of the brain activity associated to each mode.

We consider that two main reasons may have led to the lack of implementation of stochastic methods: (1) having a stochastic model that is flexible and able to capture the variance of the population (i.e., does not underfit) requires training data that captures the heterogeneity of individuals with brain injuries, which might be difficult to obtain [178]; and (2) the difficulty of getting robust stochastic models hinders their application in commercial exoskeletons, as regulatory bodies impose strict safety standards to validate such devices for clinical use [179].

Exoskeletons and prosthesis share similar challenges in terms of human–robot synchronization, but in the case of prosthetic devices, the tendency to apply stochastic methods is higher than using threshold-based approaches [180, 181]. This might be explained by the homogeneity in the gait of amputees compared to the heterogeneity observed in individuals with brain injuries [182–184]. Nevertheless, as in the case of lower-limb exoskeletons, there is a lack of use of stochastic methods in commercially available prostheses [185].

We have not found any exoskeleton in the framework of this review that implements algorithms that automatically adapt the threshold values or model parameters related to gait event identification algorithms. Gait state detection methods with the ability to adapt to diverse walking conditions, e.g., different cadences [186], are still pending to be implemented and validated on exoskeletons for individuals with brain injuries.

The most common metric used to detect gait events was the vertical ground reaction force (62.8% for all the pathologies, 60.5% for stroke and 50.0% for CP), probably due to its simplicity in the theoretical and practical implementation [187]. Ground reaction forces are directly related with the physics of foot-ground interaction. The normal or vertical force component is the one that allows to identify the phases of the foot contact and lift. Force-sensing resistors, placed at particular foot locations—e.g., heel, toe, and first and/or fifth metatarsals—were generally used to measure this metric [92, 107, 157–159, 162, 164, 177, 188–203]. Alternatively, instrumented treadmills were employed to measure anterior-posterior ground reaction forces to determine the timing of the ankle plantarflexion assistance [133, 204]. However, the suitability of this metric to treat individuals with brain injuries is questionable due to their irregular center of pressure trajectory along a walking cycle. The lack of uniformity might come from equinovarus deformity [205], excessive hip external rotation [16, 206], or reduced propioception [207, 208]. Thus, it might be challenging to develop robust gait event detection algorithms that use ground reaction forces for this specific population.

Human–robot interaction forces have only been implemented on two exoskeletons (4.6%). In the first exoskeleton, the human–robot interaction forces were employed to feed a threshold-based algorithm to detect the swing phase [107], while in the second exoskeleton they were used as the teaching signal of a pool of adaptive oscillators [161]. Only a few devices used human–robot interaction forces as control inputs [103, 107, 109, 190, 209], which might explain the scarce use of this metric in exoskeletons for individuals with brain injuries. The mechanical adaptations needed on the exoskeleton’s structure to install a force/torque sensor might also explain why the measurements of human–robot interaction forces as control inputs are not commonly used.

Only a few reviewed studies incorporated biosignals as metrics in their human–robot synchronization algorithms (4.6% of the exoskeletons for all the pathologies). For example, EEG was used by only one exoskeleton [109] to detect different locomotion modes, i.e., stop, normal walk, acceleration, and deceleration. Problems related to EEG analysis, such as feature extraction and artifact removal [58, 210, 211], might make the implementation of reliable control strategies a challenge. Furthermore, EEG-based synchronization might require high levels of attention from the patient, which might result in mental fatigue [212], and thus, might limit the training duration. Nevertheless, brain activity might be especially useful for individuals who suffer from a severe neurological condition, such as paraplegia [213, 214].

In people who preserve their voluntary muscle control over the affected limbs, muscular activity might be a more suitable metric compared to brain activity. Yet, only two devices [108, 159] validated muscular activity as an event detection metric in individuals with brain injuries. These devices employed muscular activity (EMG) from the trunk, hip, and knee flexor/extensor muscles to trigger the control action. There are several limitations associated with the use of muscular activity to detect gait events. First, surface electromyography (sEMG) signals suffer from non-robustness due to patient-to-patient variability and sensor-placement dependency [38, 59]. Moreover, muscular activity might not be reliable in individuals who have abnormal muscle activation patterns, such as stroke and CP survivors [58, 215].

We consider that joint or body segment kinematics (used in 41.8% of the exoskeletons for all the pathologies, 36.8% for stroke, and 40.0% for CP) might be more reliable metrics than the aforementioned metrics in previous paragraphs for the human–robot synchronization algorithms when detecting events with brain-injured people [216], as they show higher homogeneity among individuals with hemiplegic gait [217, 218]. In particular, the shank absolute angle and angular velocity in the sagittal plane have been shown to be especially robust metrics to detect gait events in individuals with hemiplegic gait [219].

#### Mid-level control

Trajectory-tracking control is the most used Mid-level control strategy in lower limb exoskeletons for rehabilitation (97.7%). The most common approach is to enforce predefined reference position or torque trajectories defined based on data of unimpaired joints [69, 192, 220]. Trajectory-tracking control was combined with compliant control (28.9% of the exoskeletons for stroke and 20.0% of the exoskeletons for CP) in assistive controllers based on potential [14, 107, 176, 190, 221] or velocity fields [142]. In these examples, the assistive action of the exoskeleton varied based on the joint kinematic errors.

Only four devices (13.9% of the exoskeletons) that used myoelectric control were validated on individuals with brain injuries [66, 133, 159, 204]. Myoelectric control is one of the least often employed Mid-level control strategies in post-stroke (10.5% of the exoskeletons) and cerebral palsy (10.0%) rehabilitation, according to the results of this review. The aforementioned issues with muscle activity recording and analysis (see “High-level control: human–robot synchronization” for a detailed discussion) might be behind the low adoption of this Mid-level control technique. Nonetheless, myoelectric control has a high applicability for people who preserve volitional control of the muscles, such as users of robotic prosthetic devices [222].

None of the reviewed studies incorporated BCI control with individuals with brain injuries. Problems related to the extraction of relevant information from, e.g., EEG recordings (see “High-level control: human–robot synchronization” for a detailed discussion) might also explain the lack of usage of this Mid-level control technique in exoskeletons for individuals with brain injuries. EMG is a viable alternative or adjunct to EEG for detecting movement intention or generating control signals, but the practical benefits of using EMG over EEG, e.g., shorter set-up time, more compactness, and lower doning/offing times, might explain why myoelectric control has been more often used than BCI control [214]. Few studies, aside the ones included in this review, evaluated the feasibility of using EEG signals for BCI control of exoskeletons for individuals with brain injuries [223, 224].

### Clinical validation

This section provides an overview of the most important characteristics of the clinical validation of the robotic interventions, i.e., participants’ demographics, protocol design, and outcome measures. The results summarized in this section only incorporate participants who tested the exoskeletons and not participants in the control group. See Additional file 1 to have a more detailed description about the studies included in the clinical validation.

#### Participants’ demographics

**Fig. 5:**
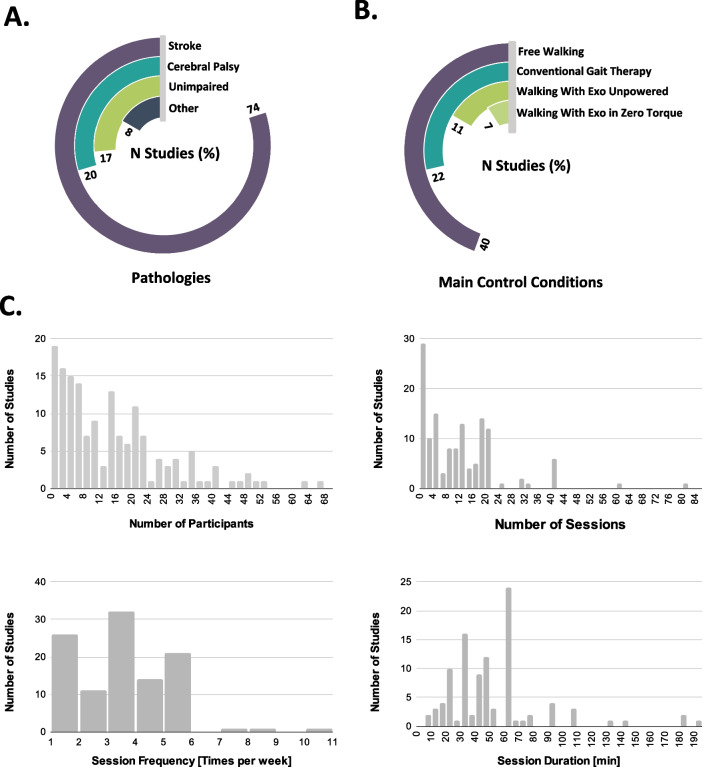
Overview of the participants’ demographics and experimental protocol characteristics. **A** Percentage distribution of the pathologies of the participants included in the reviewed studies. **B** Percentage distribution of main control conditions in the studies. **C** Histograms of number of participants (top left), number of sessions (top right), "session frequency" (times per week; bottom left), and session duration (bottom right) across the selected studies

Stroke was the main pathology of the participants recruited for the studies included in this review (74% of the studies) (see Fig. 5A). The majority of the participants with stroke were in the chronic phase (55.41% of participants with stroke), followed by subacute (33.83%) and acute (10.76%) phases. Cerebral Palsy was included in only 20% of the studies, while the representation of other brain injuries, like traumatic brain injury (1.2%) or other acquired brain injury (1.88%), was scarce. It is especially remarkable that despite the high incidence of traumatic brain injury, only two studies focused on this specific population [225, 226].

#### Experimental protocol

High variability was found in the number of participants (${14.87 \pm 13.53}$), number of sessions (${11.77 \pm 12.20}$), session frequency (times per week; ${3.09 \pm 1.68}$), and session duration (${50.57 \pm 34.06}$ min) (see Fig. 5C). Previous reviews that analyzed the protocol of robotic treatments reported similar high variability [40, 46]. Some studies did not provide complete information about the experimental protocol, e.g., they did not mention the number (15.09%), duration (33.33%), or frequency (31.44%) of the training sessions.

Free walking without the exoskeleton was the condition most often employed to compare the robotic treatment with (39.62%) (see Fig. 5B). There were also studies that compared the robotic treatment with conventional gait therapy (22.01%), while other studies compared the robotic treatment with the effect of using the device unpowered (10.69%) or in zero torque mode (6.92%).

The average level of evidence of the studies included in this review was low. The majority of the studies were observational (66.04%), while only 10.06% and 22.64% were CTs and RCTs, respectively. Only 12.58% of the studies did a follow-up evaluation after the robotic intervention, on average four months after the last intervention.

#### Outcomes of interest

**Fig. 6:**
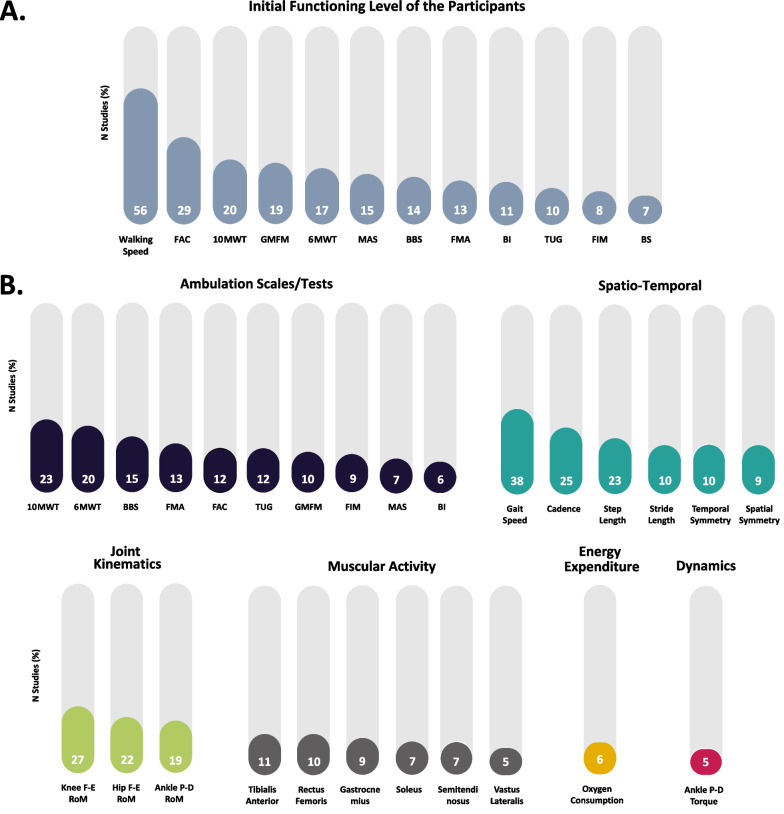
Overview of the main baseline and outcome metrics. **A** Percentage distribution of the metrics that were used in at least 5% of the studies to determine the initial functioning level of participants. **B** Percentage distribution of the outcome measures of the robotic interventions that were used in at least 5% of the studies grouped by categories, i.e., ambulation scales/tests, spatio-temporal measurements, joint kinematics, muscular activity (EMG), dynamics, and energy expenditure. Functional Ambulation Category (FAC), 10 m Walk Test (10MWT), 6 min Walk Test (6MWT), Gross Motor Function Measure (GMFM), Modified Ashworth Scale (MAS), Berg Balance Scale (BBS), Fugl–Meyer Assessment (FMA), Barthel Index (BI), Timed-Up and Go (TUG), Functional Independence Measure (FIM) and Brunnstrom Stage (BS)

Ambulation scales were the main metrics used to classify the initial functional level of participants for all the studies. The participants’ baseline was determined using metrics that analyzed their level of impairment and motor function—GMFM (19.50%), FMA (13.21%), and Brunnstrom Stage (BS) (7.55%)—, mobility—TUG (10.06%), FAC (29.56%), BBS (14.47%)—, spasticity—modified Ashworth scale (MAS) (15.09%)—, and functional capacity and activities of daily living—walking speed (56.6%), 10MWT (20.75%), 6MWT (16.98%), FIM (8.81%), and Barthel Index (BI) (11.32%) (see Fig. 6A).

A critical limitation we encountered when comparing robotic treatments was the low homogeneity across studies in the selected outcome measures after the treatment, as no metric was used in more than 50% of the studies (see Fig. 6B). Ambulation scales together with spatio-temporal parameters were similarly used to determine the effect of the robotic treatment (62.89% of the studies). Within these families of metrics, gait speed was the most used metric in the reviewed studies (37.74%), followed by cadence (25.16%) and step length (23.27%). Joint kinematics was also often used to quantify the effect of the robotic intervention (44.65%). Hip (22.64%), knee (27.67%), and ankle (18.87%) ranges of motion (RoM) in the sagittal plane were the most often selected kinematic metrics.

Finally, the number of studies that analyzed the muscular activity through sEMG was lower in comparison with the aforementioned families of metrics (20.75%). The main analyzed muscles were the ankle dorsiflexor (tibialis anterior, 10.69%) and plantaflexor (gastrocnemius, 8.81%; and soleus, 6.92%) muscles, and the knee extensor (rectus femoris, 9.43%; and vastus lateralis, 5.03%) and flexor (semitendinosus, 6.92%) muscles. Less frequently employed metrics include those related to gait dynamics (18.23%, where the most used was ankle torque in 5.03% of the studies)—i.e., joint torques and ground reaction forces, energy expenditure (10.69%, where the most used was oxygen consumption in 5.66% of the studies), and neural activity, i.e., brain activation and cortex excitability (6.29%).

### Clinical comparison of the control strategies

This section quantifies the relation between the control strategies and the clinical metrics presented in “Outcomes of interest for the clinical comparison” section to compare among strategies.

A total of 12 control strategies were evaluated in this section in terms of training intensity (min/week) and percentage of improvement of the outcome metrics selected. We considered that the most efficient control strategy for the metric analyzed would be the one that results in the highest improvement with the lowest training intensity. We also evaluated the grade of evidence—i.e., high, mid and low—and the number of participants and studies.

Based on the analyzed studies, we could only extract moderate conclusions from the studies that included post-stroke participants. The studies that involved patients with other brain injuries such as CP or traumatic brain injury did not allow for a comparison of the control strategies implemented, due to the lack of studies with exoskeletons using different control strategies. See “Limitations and future steps” section for more details.

**Fig. 7:**
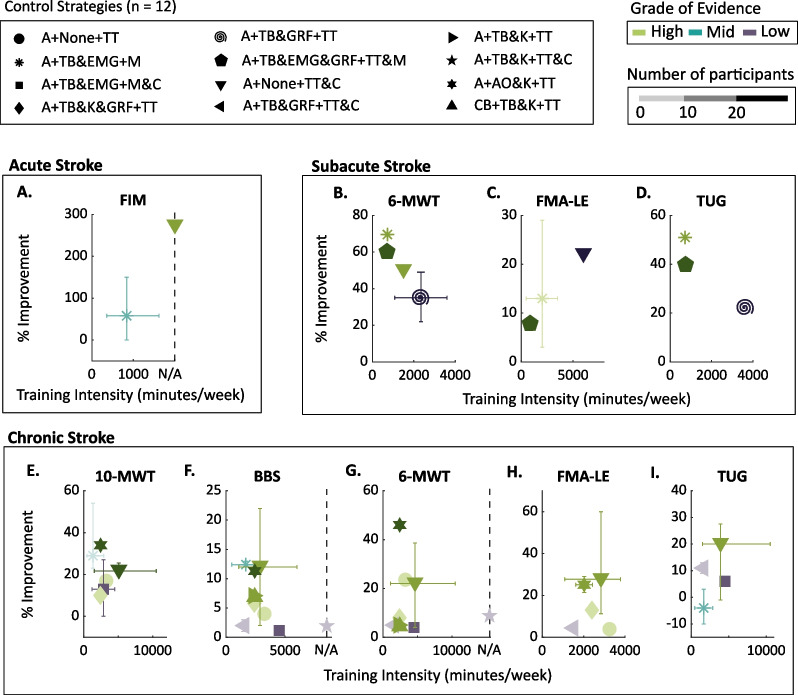
Clinical comparison of the control strategies per outcome metric and acuity level of stroke. Relation between the training intensity and percentage of improvement for **A** acute, **B**–**D** subacute and **E**–**I** chronic stroke for the selected outcome metrics. The shape of each symbol corresponds to each of the control strategies, the color is related to the grade of evidence and the intensity of the color is associated to the number of participants of the studies included. The error bars indicate the range of values for the training intensity (horizontal lines) and range of percentage of improvement (vertical lines). The control strategies included are combinations of (i) Control aim: Assistive (A), Challenge-Based (CB); (ii) Human–Robot Synchronization: Threshold-Based (TB) and Adaptive Oscillator (AO), with metrics Ground Reaction Forces (GRF), Electromyography (EMG), and Joint Kinematics (K); (iii) Mid-Level Control: Trajectory Tracking (TT), Compliant (C), Myoelectric (M). Other acronyms: Not Available (N/A), 10 m Walk Test (10MWT), 6 min Walk Test (6MWT), Berg Balance Scale (BBS), Fugl–Meyer Assessment of Lower Extremity (FMA-LE), Functional Independence Measure (FIM), and Timed-Up and Go (TUG)

As a general introductory comment to the results, all the control strategies evaluated provided a positive effect on the selected outcomes of interest for participants with stroke (see Fig. 7). Only one control strategy, i.e., assistive control with a threshold-based approach using EMG as detection metric and control signal, provided a negative impact on chronic participants for the TUG test [227, 228]. See Additional file 3 for a detailed table of the control strategies implemented in the reviewed studies and the results obtained in the main outcomes of interest for individuals with stroke.

#### Acute stroke

From the originally listed outcome metrics of interest, FIM was the only metric that allowed a comparison of the effectiveness of different control strategies in acute stroke rehabilitation [134, 229–232]. The participants included in the considered studies (${35.80 \pm 22.07}$ participants) presented an average initial FIM score of ${2.50 \pm 1.29}$ and an average training intensity of the robotic intervention of $840\;[360, 1620]$ min/week.

Assistive control strategies that implemented a combination of trajectory-tracking and compliant Mid-level control showed an improvement after training of 272.73% in FIM [229] with a strong grade of evidence (see Fig. 7A). Conversely, assistive strategies that included a threshold-based algorithm based on EMG recordings as detection metric and control signal showed a lower improvement after training of $58.33\;[0.00, 150.00]\%$ in FIM with moderate grade of evidence [134, 230–232].

However, this comparison is based on partial information as in [229] authors did not report the frequency of the sessions. We could deem that the observed higher improvement in FIM in the compliant assistive control strategies could also be explained by the longer training duration ($\approx$ 600 min) compared to the duration of training with neuromuscular assistive strategies ($\approx$ 240 min). Thus, there is not a control strategy that is clearly better than others to improve the patients' functional status (based on the FIM assessment) for acute stroke.

#### Subacute stroke

The metrics analyzed in studies with people in the subacute phase after stroke focused on: motor function (FMA-LE) [233–237], gait endurance (6MWT) [69, 234, 236, 238, 239], and general mobility (TUG) [234, 236, 238]. The initial scores of the outcomes of interest that allowed for comparison between different control strategies were on average: FMA-LE = ${18.87 \pm 3.75}$, 6MWT = ${114.45 \pm 40.77}$ m and TUG = ${29.42 \pm 10.2}$ s. The number of participants and the training intensity were on average ${26.31 \pm 17.83}$ and ${3103.63 \pm 3059.54}$ min/week, respectively.

The results presented in Fig. 7B–D pointed out that neuromuscular assistive control strategies outperformed trajectory tracking and compliant control strategies when evaluating the outcomes of interest. In particular, neuromuscular assistive control strategies that incorporated a threshold-based algorithm using EMG as the detection metric and control signal provided the highest improvements in all outcome measures with a high level of evidence [233–235]. Importantly, this type of control showed similar or higher improvements with lower training intensity and higher grade of evidence (high level) in 6MWT (69.59%; see Fig. 7B), FMA-LE (12.66% improvement; see Fig. 7C) and TUG (50.74%; see Fig. 7D), compared to the other control strategies implemented in other studies. However, the average number of participants (8 participants) was smaller than in other studies (25.5 participants), which reduces the impact of this result.

Only one study combined two different control strategies separately on the same robotic treatment [236]. In particular, the authors combined EMG and assistive control with a trajectory-tracking (Mid-level control) that used a threshold-based synchronization algorithm with ground reaction forces as input data. When compared with other control strategies from other studies, the combination of the two control strategies in Watanabe et al. [236] reached similar improvements with lower training intensity and higher grade of evidence in 6MWT (60.39%; see Fig. 7B), FMA-LE (8.42%; see Fig. 7C), and TUG (39.57%; see Fig. 7D).

In the high-evidence study from Watanabe et al. [236], the results of myoelectric control were boosted when combined with a control strategy that did not use muscle activation. As we mentioned in “High-level control: human–robot synchronization” section, myoelectric control suffers from several technical limitations when employed in individuals with abnormal muscle activation patterns. Thus, it is possible that alternative detection metrics (e.g., based on lower-limb kinematics) and Mid-level control strategies (e.g., trajectory-tracking with compliant control) might produce higher improvements with shorter training time in subacute stroke participants [240–244].

Aligned with the aforementioned comments, assistance provided by trajectory tracking and compliant control showed the highest improvement for the FMA-LE (21.95%; see Fig. 7C) for a high number of participants (38 participants). Nevertheless, the training intensity was higher and the grade of evidence was lower than the other studies.

#### Chronic stroke

Studies on people in the chronic phase after stroke were the only ones that used all the metrics described in “Outcomes of interest for the clinical comparison” section. The mean baseline values (i.e., baseline condition) were: 6MWT = ${197.03 \pm 58.53}$ m [160, 168, 171, 190, 245–254], 10MWT = ${0.42 \pm 0.23}$ m/s [160, 168, 171, 227, 228, 245, 248, 255–258], BBS = ${44.00 \pm 6.94}$ [160, 168, 171, 190, 227, 228, 245, 246, 250, 252–254, 259–261], TUG = ${31.85 \pm 20.00}$ s [160, 190, 245, 248, 249, 252, 258–260], and FMA-LE = ${37.26 \pm 53.80}$ [160, 168, 170, 171, 190, 247, 249, 250, 252, 257]. The average number of participants per study and the mean training intensity were ${16.95 \pm 12.80}$ and ${3414.31 \pm 3518.10}$ min/week, respectively.

Assistive control together with adaptive oscillators that use lower-limb kinematic information to synchronize the robot with the patient's motion and with a trajectory-tracking control as Mid-level control, achieved the best results in general (see Fig. 7E–H). Robotic treatments using this strategy showed higher or similar improvements—i.e., improvement of 46.00% in 6MWT (see Fig. 7G), 34.00% in 10MWT (see Fig. 7E), $25.19\;[21.40, 28.99]\%$ in FMA-LE (see Fig. 7H), and 11.30% in BBS (see Fig. 7I) after the treatment—with lower or similar training intensity ($2025\;[1620, 2430]$ min/week) and higher grade of evidence, compared to other control strategies implemented in other studies [168, 170]. Yet, despite being high-evidence studies with a moderate number of participants (19 participants), there were only two studies that supported the efficacy of this control strategy.

Similar improvements in the 10MWT and BBS (28.82 [9.76, 53.85]%; see Fig. 7E and 12.39 [11.82, 12.96]%; see Fig. 7F, respectively) were observed when assistive controllers with a threshold-based approach using EMG as detection metric and control signal were employed [227, 228, 255]. However, the grade of evidence and the number of participants were lower in comparison to the aforementioned studies that implemented controllers that were not neuromuscular-based. Furthermore, this type of controller was the only one that had a negative effect on the TUG score ($-3.61\;[-10.27, 3.06]\%$; see Fig. 7I) [227, 228].

Control strategies that implemented assistance in combination with trajectory tracking and compliant control showed the highest increase in TUG ($20.43\;[12.5, 41.30]\%$; see Fig. 7I) and FMA-LE ($27.76\;[11.30, 60.00]\%$; see Fig. 7H), with a strong grade of evidence [247–250, 257–260]. Nevertheless, these studies also involved the highest training intensity. Therefore, the superior improvement might be related not only to the control strategy employed, but also to the higher training intensity ([1080, 10500] min/week); in comparison with the studies that used different control strategies and that also evaluated these metrics ([480, 4500] min/week).

Only one study evaluated resistive control strategies in people in the chronic phase of stroke [253]. The authors reported an improvement in 6MWT (5.00%; see Fig. 7G) and BBS (7.14%; see Fig. 7F), which are similar to the ones reported for assistive control. Based on this, we advocate that more studies implementing resistive control strategies need to be carried out to provide stronger evidence on their clinical effectiveness.

## Discussion

The main contribution of this systematic review is that it provides a classification of the control strategies implemented on lower-limb exoskeletons, analyzes the experimental methodology used in the robotic interventions, and compares the clinical effectiveness of the control strategies when used—together with the exoskeleton—as a gait rehabilitation tool for individuals with stroke. In the following subsections, we answer to the posed three research questions of this review.

### Which control strategies have been used on powered lower limb exoskeletons for individuals with brain injuries?

Regarding the implementation of High-level controllers, we found that *assistive control* strategies are the most widely implemented on lower-limb exoskeletons for individuals with brain injuries. Despite the potential of adaptive control (see “Neuroscience evidence behind current control developments”), most of the controllers included in this review did not adapt the control parameters based on meaningful biomechanical metrics, such as hip hiking or circumduction. Thus, it is an open question whether adaptive controllers would potentially outperform current solutions. Comprehensive studies analyzing the effect of the exoskeleton control parameters on clinically meaningful biomechanical metrics might allow the development of adaptive control rules that directly tackle the main gait abnormalities of individuals with brain injuries [97, 262, 263].

As for human–robot synchronization, we found that *threshold-based techniques*, which rely on ground reaction force as detection metric, are extensively used. Only a few devices used *adaptive oscillators* to synchronize the motion of the exoskeleton with that of the patient. Yet, adaptive oscillators seem to have a high potential for this specific population. As an interesting result, only one device included in this systematic review implemented *stochastic methods* for human–robot synchronization, despite their popularity in research and potential application in identification and classification of states and actions of the human–robot system. In recent years, novel approaches have been proposed that estimate biological joint torques using musculoskeletal modelling to control the action of the exoskeleton in a state-independent manner, i.e., with no need to detect gait events or different walking conditions, e.g., stair ascent and descent [143, 264, 265]. However, these control strategies still need further investigation to evaluate their potential clinical effectiveness on individuals with brain injury.

For the Mid-level control, *position trajectory-tracking* control was the most commonly used strategy, which was combined in some cases with *compliant control* to dynamically relate joint angles to forces or torques. We consider that this approach might be the most appropriate for devices that provide partial assistance, as it promotes a dynamic synergy between the patient and the device. Only a few devices implemented *myoelectric control*, while none of them employed BCI to control lower-limb exoskeletons in this population. We attribute this shortage to the difficulty of developing generalized control laws that use EMG or EEG as control signals for individuals with brain injuries.

### What are the experimental protocols and outcome metrics used for the clinical validation of robotic interventions?

We found a wide heterogeneity in the experimental protocols and the selection of the outcomes of interest to evaluate the robotic interventions. Walking speed was the preferred metric to evaluate the patients' initial impairment level and the effectiveness of the robotic treatment. Almost all studies included in this review focused on testing the exoskeletons on participants with stroke and CP. Other types of brain injury represented a low portion of the reviewed studies.

Regarding the outcome metrics used, we consider that the field should not continue focusing solely on performance-based outcome metrics such as walking speed over a certain distance. Standard clinical metrics have been useful in the past years to quantitatively evaluate the progress of impaired individuals. However, they might fail in showing the specific biomechanical effects of the treatments. For example, participants could have a better score on the 10MWT after training, i.e., walk faster, but they might use more compensatory strategies, e.g., hip hiking or circumduction, or a stronger involvement of the non-impaired leg. This does not necessarily have to be a negative circumstance, but the 10MWT alone does not allow to link a potential effect of the robotic training to the underlying (biomechanical) mechanism through which the improvement is achieved.

Thus, we consider that using biomechanical metrics, which are more directly related to the impairment itself, could complement standard clinical outcomes by providing a more detailed perspective of the effect of the robotic treatment. Moreover, improvements in these biomechanical metrics might also result in better scores in the standard clinical tests as they are related. Finally, biomechanical outcomes, e.g., step length or temporal symmetry ratio, can be used independently of the level of impairment. In fact, there are some clinical tests, e.g., the 6MWT, that are quite difficult to be carried out by participants with high levels of impairment. In this literature survey, we have found that the number of studies that used outcomes directly related to gait disorders is increasing. However, those studies included a wide range of biomechanical metrics that did not allow a comparison among studies. For instance, step length, which is one of the quantifiable markers of impaired walking performance and motor recovery, was used only in 23% of the studies. Future research could focus on identifying a core set of biomechanical outcome metrics to be used in prospective trials.

As complementary results to the biomechanical outcomes, we think that reporting information about the set-up timings and the level of participation of the patient through the treatment might provide relevant information to compare control strategies. None of the studies included in this review reported set-up times of the control parameters of the exoskeletons. Yet, the time to tune the control parameters might be a relevant point to consider when comparing control strategies. This is important because during practice therapists have to tailor the device for multiple patients in a reduced time frame. Fortunately, recent publications of exoskeletons tested on individuals with brain injuries have started to include this metric [100, 101]. Furthermore, we did not find any study that reported metrics that might be directly associated with the level of participation of the patient through the robotic treatment, e.g., direct measurements of muscle activation or indirect estimation through the reporting of control parameters. For example, reporting the value of the adapted control parameters along the training might provide an estimate of the patients’ walking ability [266, 267].

The variety in the experimental protocols and the reported performance metrics are the main factors which hinder a systematic comparison between the controllers effectiveness. We think that the same outcomes and experimental protocol have to be used for studies in which the participants have the same pathology and level of impairment, so it is possible to compare among control strategies. Furthermore, studies with exoskeletons that target the same joints should use the same experimental methods to allow for hardware-independent comparisons among control strategies.

### What is the current clinical evidence on the effectiveness of the different control strategies?

We were unable to identify a control strategy that is clearly superior for acute stroke patients. Assistive control strategies that implemented a combination of trajectory-tracking and compliant control showed the highest clinical effectiveness, with high grade of evidence and a moderate number of participants (19 participants), but they also required the longest training time. Assistive control strategies that followed a threshold-based algorithm with EMG as detection metric and control signal provided the highest improvements with the lowest training intensities and low number of participants (8 participants) in the outcome measures of interest for subacute stroke. Finally, adaptive oscillators that used lower limb kinematic information to assist the motion of the user together with trajectory-tracking as Mid-level control showed the highest improvements with reduced training intensities for chronic stroke with high evidence (all RCT) with a moderate mean number of participants (19 participants). Finally, we were not able to determine the efficacy of adaptive control strategies as none of the studies that implemented these strategies fullfilled the inclusion criteria for the clinical comparison.

Note that these conclusions should be treated with the consideration that the number of studies for the clinical comparison of the control strategies was low. A total of 73 studies were included in the comparison (see Fig. 1), but our conclusions are based on only 57 studies. Nevertheless, the majority of these studies are of high quality (see Additional file 3 for detailed information about the quality of the studies for each family of control strategies). Thus, there is a trade-off between quality and quantity. On the one hand, we consider that results are fairly consistent across the studies and come from high-level evidence studies with large sample sizes, i.e., RCTs or CTs, which minimizes the risk of bias. On the other hand, we think that the results would be stronger if treatments included a larger aggregate pool of participants and/or with wider inclusion criteria, which would allow the generalizability of the outcomes of the studies to a broader population.

### Limitations and future steps

Although the number of studies that evaluated the effectiveness of robotic-assisted gait rehabilitation has increased exponentially in the last decade, we still found critical limitations in the clinical comparison of the effectiveness of different control strategies. Only a few studies compared control strategies on the same participants and using the same exoskeleton, hindering the possibility to extract clear conclusions regarding the clinical effectiveness of each control strategy for gait rehabilitation. In addition, spontaneous recovery [69] and compensation strategies probably contributed to increased scores on the outcome metrics, making it challenging to purely evaluate the effect of the different control strategies on functional recovery among different studies.

Another relevant limitation is that our comparison was limited to individuals with stroke. We were not able to evaluate control strategies of studies that involved patients with CP or traumatic brain injury, due to the lack of studies with exoskeletons using different control strategies and the heterogeneity of the level of the impairment of the participants. For the case of CP, in the studies that reported the main outcomes of interest, participants were pooled together, independently of their GMFCS level [268–271]. Only in a few studies that used the Lokomat [272–276] and CPWalker [277], authors analyzed the outcomes of interest selected in “Outcomes of interest for the clinical comparison” section and differentiated between the GMFCS levels. However, those studies implemented the same family of control strategies, namely assistive control strategies without human–robot synchronization algorithms that combined trajectory-tracking and compliant control, and thus, no comparison between controllers was possible.

**Fig. 8:**
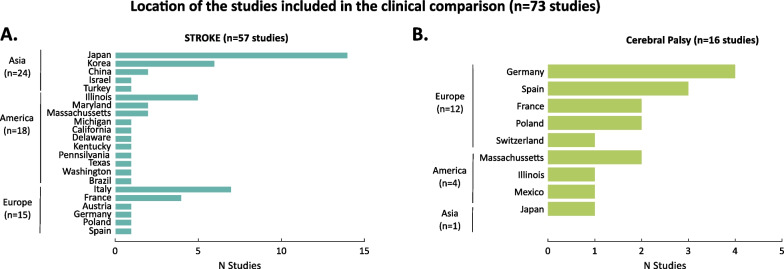
Location of studies included in the clinical comparison. **A** Number of the studies per location for participants with stroke. **B** Number of the studies per location for participants with cerebral palsy

While the level of evidence of the studies included in the clinical comparisons is high, the number of studies for each family of control strategies is still low. The reduced number of studies might be a consequence of the regulatory framework for medical devices, which limits the opportunity of validating the technology at early stages of development (see Fig. 8 for the geographical location of the studies included in the clinical analysis). With current tight regulations, testing devices at a low Technology Readiness Level (TRL) is subject to the same requirements as those devices that are ready to be certified [111, 179, 278]. Furthermore, there is a lack of an ethical and regulatory framework that enables researchers to involve end-users in the co-creation and validation of early-stage prototypes to quickly make technology accessible to the users, while guaranteeing the well-being of patients and therapists.

## Conclusion

This paper presents one of the first reviews that focuses on the effectiveness on rehabilitation of different control algorithms used in lower limb exoskeletons for gait rehabilitation after brain injury. This literature survey is a first step towards determining the most effective control algorithms for each pathology and level of impairment. The main findings from this review are: (1) We found that assistive controllers that followed threshold-based algorithms relying on ground reaction force thresholds in conjunction with trajectory-tracking control were the most implemented control strategies. Few devices implemented adaptive control strategies that modulated the control parameters based on the patients’ performance. (2) Aligned with other reviews on clinical practice of robotic interventions, we found high variability in the experimental protocols and selected outcome metrics. (3) Assistive control strategies that implemented a combination of trajectory-tracking and compliant control showed the highest clinical effectiveness for acute stroke. Assistive control strategies that followed a threshold-based algorithm with EMG as detection metric and control signal provided the highest improvements in the outcome measures of interest for subacute stroke. Assistive control strategies, which followed threshold-based or adaptive oscillator algorithms together with trajectory-tracking control, resulted in the highest improvements for individuals with chronic stroke. For other brain injuries included in this review—i.e., cerebral palsy and traumatic brain injury—the lack of standardization on the clinical studies made it impossible to analyze the effect of the control strategies on the clinical outcomes of interest.

Although remarkable efforts have been made into developing novel sophisticated motor-learning driven controllers to enhance gait rehabilitation, the majority of the reviewed studies only provided a general overview of the effect of the robotic controller on individuals with brain injuries. Future research should evolve into structured and standardized studies that aim at finding the relation between control strategies and a core-set of clinical outcome measures, controlling for the effects of participants’ initial impairment level and training intensity. Current limitations might be overcome when clinicians, researchers, industry, and regulatory bodies work together to solve this urgent societal and scientific problem.

## Supplementary Information

**Additional file 1.** Analysis of the studies included in the review.

**Additional file 2.** Table with the studies included in the clinical comparison.

**Additional file 3.** Relation between outcome metrics and control strategies for stroke.

## Data Availability

All data generated or analysed during this study are included in this published article and its Additional files.
